# Clinical Implications of Circulating Tumor Cells in Patients with Esophageal Squamous Cell Carcinoma: Cancer-Draining Blood Versus Peripheral Blood

**DOI:** 10.3390/cancers16162921

**Published:** 2024-08-22

**Authors:** Dong Chan Joo, Gwang Ha Kim, Hoseok I, Su Jin Park, Moon Won Lee, Bong Eun Lee

**Affiliations:** 1 Department of Internal Medicine, Pusan National University School of Medicine, Busan 49241, Republic of Korea; asclllepios@pusan.ac.kr (D.C.J.); neofaceoff@hanmail.net (M.W.L.); bongsul@hanmail.net (B.E.L.); 2 Biomedical Research Institute, Pusan National University Hospital, Busan 49241, Republic of Korea; soo-jean@hanmail.net; 3 Department of Thoracic and Cardiovascular Surgery, Pusan National University School of Medicine, Busan 49241, Republic of Korea

**Keywords:** circulating tumor cells, esophageal neoplasms, squamous cell carcinoma, azygos vein, TWIST

## Abstract

**Simple Summary:**

The levels of circulating tumor cells (CTCs) in blood from cancer-draining veins hold diagnostic and prognostic significance across various cancers. However, the research on esophageal squamous cell carcinoma (ESCC) remains limited. This study aimed to compare CTCs based on the sampling site (peripheral vein vs. cancer-draining azygos vein) and to investigate their association with the clinicopathological characteristics of patients with ESCC. The counts of CTCs and TWIST (+) CTCs were notably higher in blood from the azygos vein compared to that from the peripheral vein. However, CTCs and TWIST (+) CTCs in both peripheral and azygos vein blood did not show any correlation with clinicopathological characteristics. This study highlights the need for further research on the potential role of CTCs in azygos vein blood as predictive biomarkers for prognosis and chemotherapy responses in patients with ESCC.

**Abstract:**

Circulating tumor cells (CTCs) in cancer-draining veins have diagnostic and prognostic value. However, studies on esophageal squamous cell carcinoma (ESCC) are limited. This study aimed to compare CTCs obtained from different sampling sites (peripheral vein vs. cancer-draining azygos vein) and to investigate their association with the clinicopathological characteristics of ESCC patients. Blood samples were collected preoperatively from both veins in 40 ESCC patients at Pusan National University Hospital from June 2020 to April 2022. CTCs were detected using a centrifugal microfluidic method with fluid-assisted separation. CTCs and TWIST (+) CTCs were detected more frequently in the azygos vein blood than in the peripheral vein blood; however, the difference was not statistically significant (85.0% [34/40] vs. 77.5% [31/40], *p* = 0.250 and 82.5% [33/40] vs. 75.0% [30/40], *p* = 0.586, respectively). CTC and TWIST (+) CTC counts were significantly higher in the azygos vein blood than in the peripheral vein blood (7 vs. 3, *p* < 0.001, and 6 vs. 2, *p* < 0.001, respectively). CTCs and TWIST (+) CTCs from peripheral and azygos veins showed no association with clinicopathological characteristics. Further large-scale studies are needed to clarify their role as predictive biomarkers for prognosis and chemotherapy responses in ESCC patients.

## 1. Introduction

Esophageal cancer is the eighth most prevalent type of cancer worldwide and the sixth leading cause of cancer-related death [1,2,3]. Esophageal cancer is histopathologically divided into squamous cell carcinoma and adenocarcinoma. Esophageal squamous cell carcinoma (ESCC) accounts for over 90% of esophageal cancer cases in East Asian countries such as Korea, China, and Japan, with patients’ overall survival rate being 25–30% [4,5]. Because patients with early-stage ESCC do not exhibit specific symptoms and signs, most are diagnosed at advanced stages, characterized by the invasion of adjacent organs or distant metastases [5,6]. The stage at the time of diagnosis is a determining factor for clinical outcomes. The overall survival rate of patients with early-stage ESCC exceeds 80%; however, the 5-year survival rate of patients with advanced-stage ESCC is <20% [7]. Therefore, the early diagnosis of ESCC is essential for achieving better clinical outcomes [7,8]. However, the upper gastrointestinal endoscopy and serum tumor markers currently used have limitations in diagnosing early-stage ESCC [9,10].

Circulating tumor cells (CTCs) are cancer cells that arise from primary or metastatic cancers and travel through the bloodstream of patients with cancer [11]. During the epithelial–mesenchymal transition (EMT) process in tumor cells, epithelial markers such as the epithelial cell adhesion molecule (EpCAM) and cytokeratin (CK) are downregulated, whereas mesenchymal markers, including TWIST, are upregulated [12]. This EMT process enables tumor cells to detach from the primary or metastatic sites and enter the bloodstream through intravasation. As a result, CTCs and TWIST (+) CTCs can be detected in the venous blood of patients with various cancers [13].

Detecting CTCs in the peripheral blood of patients with various types of cancers has both diagnostic and prognostic implications [14]; nevertheless, this process remains challenging. The rarity of CTCs in the peripheral blood of patients with various malignancies, particularly those without metastasis, limits their clinical application as diagnostic and prognostic biomarkers. Therefore, trials involving blood sample collection from cancer-draining veins have been conducted for various malignancies. For example, when blood samples are obtained from the portal vein in patients with pancreatic ductal adenocarcinoma and from the pulmonary vein in those with non-small-cell lung cancer, a higher CTC detection rate is observed compared with peripheral blood sampling in each case [15,16,17]. Additionally, CTCs detected in the blood from cancer-draining veins show a more significant association with prognosis than those identified in the peripheral blood of patients with pancreatic ductal adenocarcinoma and colorectal cancer [15,18]. Nevertheless, only a few studies have investigated CTCs in the blood from the cancer-draining veins of patients with ESCC [19]. Therefore, the present study aimed to compare CTCs according to the sampling site (peripheral vein vs. cancer-draining azygos vein) and to explore the relationship between CTCs and the clinicopathological characteristics of patients with ESCC.

## 2. Materials and Methods

### 2.1. Study Population

**Figure 1:**
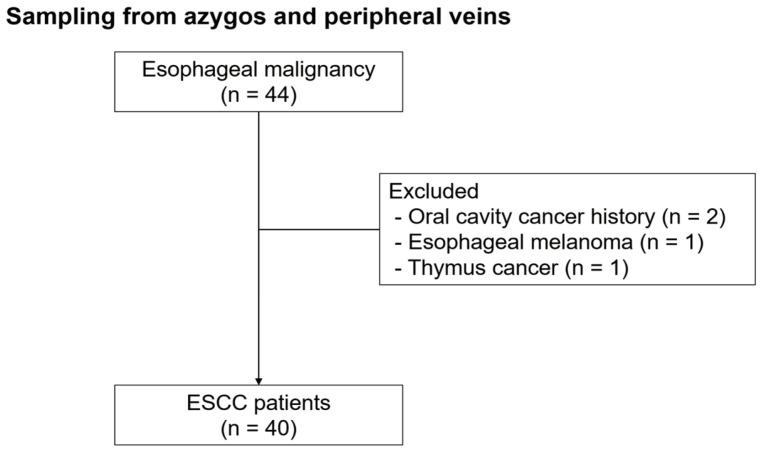
Flowchart of patients with esophageal squamous cell carcinoma enrolled in this study.

Blood samples from the peripheral and azygos veins were prospectively obtained from 44 patients with esophageal malignancy before esophagectomy between June 2020 and December 2022 at Pusan National University Hospital (Busan, Korea). Patients with a history of other malignancies within 5 years were excluded from this study. Accordingly, four patients were excluded from the analysis for the following reasons: two had a history of oral cavity cancer, one had esophageal melanoma, and one had thymus cancer. Finally, 40 patients with ESCC were enrolled in this study (Figure 1). Twelve patients in the advanced stage underwent two cycles of neoadjuvant chemotherapy (5-fluorouracil and cisplatin) before esophagectomy. These patients underwent surgery at least 4 weeks after receiving their last neoadjuvant treatment. The cancer stage was determined based on pathological findings after esophagectomy using the eighth edition of the American Joint Committee on Cancer (AJCC)’s TNM classification for ESCC staging.

This prospective study protocol was approved by the Institutional Review Board of Pusan National University Hospital (protocol code: H-1601-015-037). Written informed consent was obtained from all patients prior to blood sampling.

### 2.2. Blood Sampling from the Peripheral and Azygos Veins

**Figure 2:**
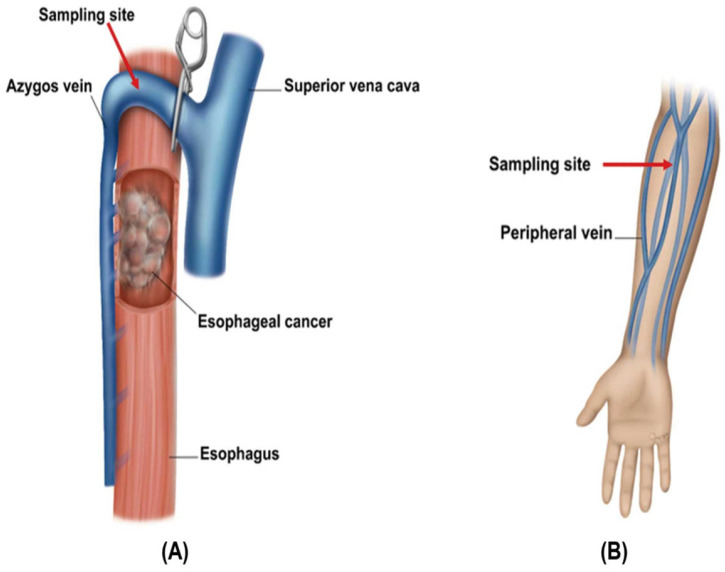
Sampling sites for circulating tumor cells in the blood from (**A**) azygos and (**B**) peripheral veins.

CTC counts in the cancer-draining vein could significantly increase after surgical manipulation of the cancer lesion [20,21,22]; therefore, all blood samples were obtained prior to the manipulation of the esophageal cancer lesion. First, 3 mL of blood was obtained from the peripheral vein in the operating room after the patient was placed under general anesthesia but before the surgical procedure began. For sampling from the cancer-draining blood, 3 mL of blood was obtained from the azygos vein after ligating the confluence of the azygos vein and superior vena cava through thoracotomy or thoracoscopy before manipulating the cancer lesion (Figure 2). Blood samples were collected in dipotassium ethylenediaminetetraacetic acid tubes and promptly inverted 10 times. The blood samples were analyzed within 8 h to prevent cell damage. This sampling protocol was strictly adhered to for all patients in this study under the supervision and operation of a single surgeon (H.I.) to ensure consistency across procedures.

### 2.3. Surgery

One highly experienced surgeon (H.I.) with over a decade of expertise performed all operations in this study. Transthoracic Ivor–Lewis esophagectomy or three-hole minimally invasive esophagectomy with two-field or three-field lymph node dissection was performed, depending on the tumor location. Furthermore, intrathoracic or cervical esophagogastric anastomosis was performed after esophageal resection.

### 2.4. Isolation and Enumeration of CTCs

Plasma was obtained by centrifuging the whole blood at 800× *g* for 10 min. To isolate a buffy coat, the blood, excluding plasma, was carefully layered on top of the Ficoll–Paque PLUS solution (GE Healthcare, Uppsala, Sweden) and centrifuged at 800× *g* for 15 min. The second white layer was extracted and washed with 10 mL of phosphate-buffered saline (PBS). The pellet was then suspended in 1 mL of red blood cell lysis buffer for 3–5 min at room temperature, followed by centrifugation at 400× *g* for 3 min. After repeated washing in PBS, the buffy coat was stored at −80 °C.

CTCs in patients with ESCC were detected using a centrifugal microfluidic system with a fluid-assisted separation technique (FAST) [13,23]. A CD-PRIME system (Clinomics, Ulsan, Republic of Korea), a commercialized version of the FAST disc, was used to isolate CTCs from the buffy coat. The disc surface was treated with 1% bovine serum albumin and rinsed with PBS before CTC isolation. The buffy coat was resuspended in PBS, using the same amount as blood required, before buffy coat separation. Subsequently, the buffy coat was injected into the disc, and CTCs were isolated from the track-etched polycarbonate membrane using a spinning disc apparatus. 

**Figure 3:**
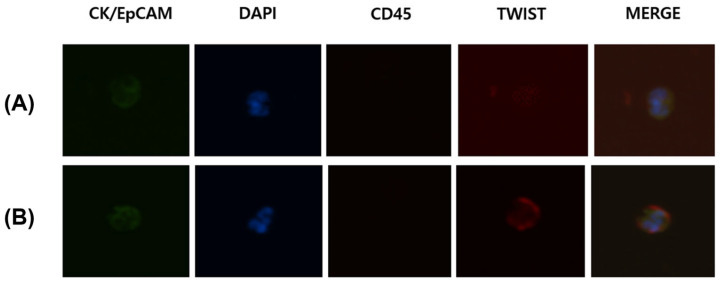
Circulating tumor cells (CTCs) were detected in the blood from the azygos veins of patients with esophageal squamous cell carcinoma. Captured cells were identified as CTCs if they were CK+ or EpCAM+, CD45−, DAPI+, and >8 μm in diameter. (**A**) Representative images of CTCs negative for TWIST immunostaining. (**B**) Representative images of CTCs positive for TWIST immunostaining. Abbreviations: CK, cytokeratin; EpCAM, epithelial cell adhesion molecule; DAPI, 4′,6-diamidino-2-phenylindole.

Immunostaining was conducted on the discs to detect CTCs within the buffy coat. Initially, the trapped cells were fixed with 4% formaldehyde for 20 min at room temperature. The fixed cells were then permeabilized in PBS using 0.1% Triton X-100 for 5 min and subsequently washed with PBS. Next, the samples were blocked with 20 μg/mL immunoglobulin G (Polyclonal Human IgG; R&D Systems, Minneapolis, MN, USA) for 20 min. Antibody staining was then performed. Anti-CD45 PE-Alexa Fluor (H130; Life Technologies, Carlsbad, CA, USA) was used to stain white blood cells for 20 min, followed by the washing of the samples with 0.01% Tween 20 in PBS. The membrane was then treated with a mixture containing fluorescein isothiocyanate-conjugated anti-CK (CAM5.2; Becton, Dickinson and Company, Franklin Lakes, NJ, USA), Alexa Fluor 488-conjugated anti-pan CK (AE1/AE3; eBioscience, Inc., San Diego, CA, USA), fluorescein isothiocyanate-conjugated anti-epithelial cell adhesion molecule (9C4; BioLegend, San Diego, CA, USA), and TWIST (Twist2Cla; BioMatrix Research, Chiba, Japan). The cells were incubated for 20 min and then washed with 0.01% Tween 20 in PBS. To detect TWIST, the Alexa Fluor 647-conjugated secondary antibody (anti-goat; Invitrogen, Carlsbad, CA, USA) was applied for 20 min and then washed. The final step involved mounting the membrane onto glass slides with a medium containing the fluorescent nucleic acid dye 4′,6-diamidino-2-phenylindole (DAPI). The CTCs on the membrane were visualized by scanning the slides under an Eclipse Ti-E fluorescent microscope (Nikon, Tokyo, Japan) at 40× magnification. Captured cells were identified as CTCs if they were CK+ or EpCAM+, CD45−, or DAPI+ and with a diameter greater than 8 μm. CTCs that were positive for TWIST immunostaining were defined as TWIST (+) CTCs (Figure 3).

### 2.5. Statistical Analysis

Continuous data are presented as median values and ranges. A Shapiro–Wilk test was performed to assess normality, and it confirmed that the data did not meet the assumption of normality. Consequently, non-parametric tests were conducted. The difference in CTC counts between the peripheral vein blood and azygos vein blood was assessed using the Wilcoxon signed-rank test. Mann–Whitney U or Kruskal–Wallis H tests were used to analyze the relationship between CTC counts and categorical clinicopathological data. The correlation between high or low CTC counts and categorical clinicopathological factors was examined using the chi-square or Fisher’s exact tests. Statistical analyses were conducted using IBM SPSS version 27.0 for Windows (IBM Co., Armonk, NY, USA), and statistical significance was set at a *p*-value <0.05.

## 3. Results

### 3.1. Baseline Clinicopathological Characteristics of Patients with ESCC

Table 1 summarizes the clinicopathological characteristics of 40 patients with ESCC. Among these patients, 35 were men and 5 were women, with a median age of 66 years (range, 51–79 years). Tumor locations were distributed as follows: two in the upper third of the esophagus, 27 in the middle third, and 11 in the lower third. The median tumor size was 3.4 cm (range, 0.6–8.6 cm). Histopathologically, 30 tumors were moderately differentiated, whereas 10 were poorly differentiated. Regarding T stage, 27 tumors were classified as T1, 6 as T2, and 7 as T3, respectively. For the N stage, 20 tumors were classified as N0, 13 as N1, 4 as N2, and 3 as N3. In terms of TNM stage, 17 patients were identified as stage I, 9 as stage II, 11 as stage III, and 3 as stage IV. Overall, 12 patients underwent esophagectomy via thoracoscopy, whereas 28 underwent esophagectomy via thoracotomy.

### 3.2. CTCs in the Peripheral and Azygous Vein Blood of Patients with ESCC

**Figure 4:**
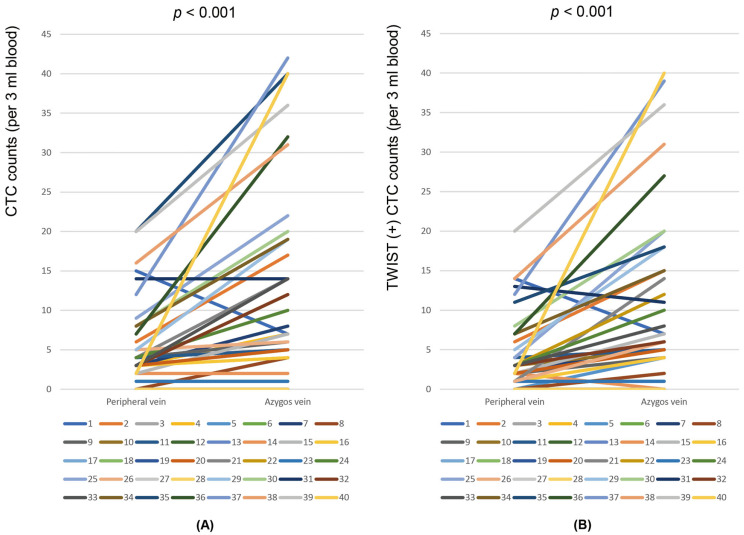
Comparison of circulating tumor cells (CTCs) (**A**) and TWIST (+) CTC counts (**B**) in blood from peripheral and azygos veins of 40 patients with esophageal squamous cell carcinoma.

CTCs were identified in the blood from the peripheral veins of 31 patients and the azygos veins of 34 patients. CTCs were detected more frequently in the azygos vein than in the peripheral vein; however, the difference was not statistically significant (85.0% [34/40] vs. 77.5% [31/40], *p* = 0.250). The median CTC count was 7 (range, 0–42) per 3 mL of blood in the azygos vein and 3 (range, 0–20) per 3 mL of blood in the peripheral vein, with a significant difference in CTC numbers observed between the two veins (*p* < 0.001) (Figure 4A). TWIST (+) CTCs were identified in the peripheral veins of 30 patients and the azygos veins of 33 patients. TWIST (+) CTCs were detected more frequently in the azygos vein than in the peripheral vein; however, this difference did not reach statistical significance (82.5% [33/40] vs. 75.0% [30/40], *p* = 0.586). The median TWIST (+) CTC count was 6 (range, 0–40) per 3 mL of blood in the azygos vein and 2 (range, 0–20) per 3 mL of blood in the peripheral vein, with the number of TWIST (+) CTCs being significantly higher in the azygous vein than in the peripheral vein (*p* < 0.001) (Figure 4B).

### 3.3. Association between CTCs and Clinicopathological Characteristics in Patients with ESCC

Table 2 presents the CTC counts in the peripheral and azygos veins according to the clinicopathological characteristics of 40 patients with ESCC. Notably, no significant differences were observed in CTC counts between the peripheral and azygos veins according to age, sex, tumor location, tumor size, histology, lymphovascular invasion, and TNM stage. Similarly, there were no significant differences in TWIST (+) CTC counts between the peripheral and azygos veins based on clinicopathological characteristics.

Patients with ESCC were categorized into low- and high-CTC groups based on the median number of CTCs and TWIST (+) CTCs present in both blood samples. Notably, there were no significant differences in clinicopathological characteristics between the low (<3 CTCs)- and high (≥3 CTCs)-CTC groups in the peripheral vein blood and between the low (<7 CTCs)- and high (≥7 CTCs)-CTC groups in the azygos vein blood (Table 3). Similarly, there were no significant differences in clinicopathological characteristics between the low (<2 TWIST [+] CTCs)- and high (≥2 TWIST [+] CTCs) TWIST (+)-CTC groups in the peripheral vein blood and between the low (<6 TWIST [+] CTCs)- and high (≥6 CTCs) TWIST (+)-CTC groups in the azygos vein blood (Table 3).

### 3.4. Subgroup Analysis for Patients Without a History of Neoadjuvant Chemotherapy

Surgery was performed at least 4 weeks after the last neoadjuvant treatment; however, the impact of neoadjuvant chemotherapy on CTCs could not be entirely excluded. Therefore, 28 patients with no history of adjuvant chemotherapy were included in the subgroup analysis. The patients were categorized into low- and high-CTC/TWIST (+) CTC groups according to the median number of CTCs and TWIST (+) CTCs in both venous samples (Table 4). TWIST (+) CTCs were associated with tumor location (*p* = 0.022); in contrast, other clinicopathological characteristics were not associated with CTCs or TWIST (+) CTCs in the blood from the peripheral and azygos veins (Table 4).

## 4. Discussion

In this study, we investigated CTCs and TWIST (+) CTCs in the blood from the peripheral and azygos veins of patients with ESCC. CTCs and TWIST (+) CTCs were identified in the blood from the peripheral veins of 31 (77.5%) and 30 (75.0%) patients and in the blood from the azygos veins of 34 (85.0%) and 33 (82.5%) patients (85.0%), respectively. The CTC and TWIST (+) CTC counts in the blood from the azygos vein were significantly higher than those in the blood from the peripheral vein. However, CTCs and TWIST (+) CTCs in the blood from the peripheral and azygos veins were not associated with clinicopathological characteristics. To our knowledge, this is the most extensive study evaluating CTCs and TWIST (+) CTCs in the blood from the azygos vein, a cancer-draining vein of esophageal cancers, in patients with ESCC.

The venous drainage system differs depending on the esophageal site. The thoracic esophagus primarily drains into the azygos vein and, to a lesser extent, the hemiazygos and intercostal veins. However, the cervical esophagus drains into the inferior thyroid vein, whereas the abdominal esophagus mainly drains into the left gastric and upper short gastric veins [24]. Consequently, given the extensive network of blood and lymphatic vessels in the submucosa of the esophagus, almost all cancers in the esophagus can drain into the azygos vein, irrespective of tumor location. In addition, there is a connection between the left gastric veins and the azygos vein system through the posterior brachial vein [24]. Therefore, the azygos vein was selected as the cancer-draining vein in this study.

In this study, the counts of CTCs and TWIST (+) CTCs in blood samples from the azygos vein were significantly higher compared to those from the peripheral vein (7 vs. 3, *p* < 0.001 and 6 vs. 2, *p* < 0.001, respectively). These findings align with a recent study on patients with ESCC, which also reported higher CTC counts in azygos vein blood than in peripheral vein blood. However, azygos vein sampling was only performed in 13 of the 88 patients in the study [19]. Compared with this study, our study used a relatively large number of blood samples from the azygos veins of patients with ESCC. Similarly, in patients with pancreatic ductal adenocarcinoma, the CTC count in portal vein blood was significantly higher than that in peripheral vein blood [15,16]. Furthermore, in patients with non-small-cell lung cancer, the CTC count in pulmonary vein blood was significantly higher than that in peripheral vein blood [17]. Additionally, in patients with breast cancer, the CTC count in cancer-draining vein blood was significantly higher than that in peripheral vein blood [25]. The higher CTC count in cancer-draining vein blood compared to peripheral vein blood can be explained by the fact that CTCs in cancer-draining blood originate directly from primary cancer lesions [26,27]. Therefore, direct blood sampling from cancer-draining veins can overcome the current limitations in the clinical use of CTCs in peripheral blood as biomarkers if possible. However, CTC counts can increase following the surgical manipulation of the cancer lesion [20,21,22]. Therefore, peripheral vein samples were collected before any surgical procedures were performed in the operating room, and all azygos vein samples were collected before any handling of the esophageal cancer lesions. 

In this study, CTCs and TWIST (+) CTCs in the blood from the peripheral and azygos veins were not associated with clinicopathological characteristics, including age, tumor location, size, and differentiation. These findings are not consistent with the results of previous studies that showed that CTCs detected in the cancer-draining vein are associated with cancer stage and lymph node metastasis in various malignancies [15,28,29,30]. The difference in our results may have stemmed from the unique characteristics of ESCC compared with other types of cancers. However, given that an association between cancer stage and CTCs has been reported in the peripheral vein of patients with ESCC [14], this discrepancy could be attributed to the inclusion of patients with ESCC who underwent neoadjuvant chemotherapy before surgery and the relatively small number of included patients. Neoadjuvant chemotherapy reduces the number of CTCs [31]. When subgroup analysis excluding the 12 patients who received neoadjuvant chemotherapy was performed, TWIST (+) CTCs were higher in the peripheral blood of patients with ESCC located in the middle third of the esophagus. 

EMT is crucial in cancer progression, metastasis, and recurrence [12]. During EMT, tumor cells gain the ability to detach from primary or metastatic sites and enter the bloodstream through intravasation. Consequently, CTCs can be detected in the venous blood of patients with various cancers, including ESCC. Among mesenchymal markers, TWIST has been implicated in ESCC progression, acting as a potential promoter of tumor invasion and metastasis [12,32]. Additionally, TWIST has been suggested as an independent prognostic marker, capable of predicting distant metastasis and survival rates in patients with ESCC [33]. Our previous study demonstrated that TWIST (+) CTCs were detected, even in early-stage ESCC, and TWIST (+) CTC count was associated with poor differentiation in patients with ESCC [13]. However, in this study, TWIST (+) CTCs in the blood from the peripheral and azygos veins were not associated with any clinicopathological factors, including tumor differentiation. These results could be attributed to the small number of cases in the present study and differences in the study population between the present study and our previous studies (only patients who underwent esophagectomy were included in the present study).

Considering the difference in the venous drainage system based on tumor location, the 40 tumors were categorized into the mid-esophagus and upper/lower esophagus groups. The CTC and TWIST (+) CTC counts in the mid-esophagus group were higher than those in the upper and lower esophagus groups; however, this difference was not statistically significant in the blood from the azygos vein (CTCs, 8.0. vs. 4.0, *p* = 0.124; TWIST [+] CTCs, 6.0 vs. 2.0, *p* = 0.106). These findings were confirmed in the peripheral vein (CTCs, 4.0. vs. 2.0, *p* = 0.308; TWIST [+] CTCs, 3.0 vs. 1.0, *p* = 0.106) (Supplementary Table S1). In a subgroup analysis of 27 patients with mid-esophageal ESCC, CTC counts in the blood from the azygos vein were associated with sex (*p* = 0.027) (Supplementary Table S2). However, these results might have been due to male predominance among the included patients.

CTCs in the blood from cancer-draining veins have some clinical implications. First, CTCs in the blood from cancer-draining veins predict the prognosis in patients with various malignancies, including colorectal, pancreatic cancer, and hepatocellular carcinoma, better than CTCs in the peripheral vein [15,18,29,34,35,36]. Second, because CTCs found in the blood from cancer-draining veins are directly derived from the primary cancer, they can better reflect the state of the primary cancer [25,37]. However, CTCs found in the peripheral blood are affected by primary and metastatic lesions [25]. Lastly, in a recent study, CTCs were also detected, even in the blood from the cancer-draining veins of patients with breast cancer who achieved pathological complete remission (CR) after neoadjuvant chemoradiotherapy [25]. Therefore, it is imperative to maintain vigilant surveillance in patients with detectable CTCs in the blood from cancer-draining and peripheral veins, even after achieving pathological CR through preoperative chemoradiotherapy.

The present study had some limitations. First, we included only patients who underwent esophagectomy for ESCC, and there were no cases of distant metastases. Second, the number of patients included in this study was small, especially the number of cases with cancers in the upper esophagus and a well-differentiated histology. Third, we focused on CTCs and TWIST (+) CTCs in the blood from the peripheral and azygos veins at the time of surgery; however, we did not investigate the long-term follow-up results, such as prognosis and survival. In future research, we will investigate the roles of CTCs and TWIST (+) CTCs, especially in the blood from the azygos vein, to predict the long-term outcomes and responses to chemotherapy. Fourth, blood sampling from the azygos vein is only possible intraoperatively, potentially limiting the advantages of liquid biopsies. However, although the detection rate of CTCs in peripheral blood was not as high as that in the azygos vein, a significant proportion of CTCs were still detected in peripheral blood. This suggests that CTCs can be evaluated in peripheral blood in the cases of ESCC that are not amenable to surgery. Additionally, unlike the azygos vein, which can only be sampled during surgery, peripheral blood can be drawn at any time, making it a valuable tool for monitoring ESCC patients over time [19]. This limitation could be overcome if other methods, such as endoscopic ultrasound-guided approaches, can be developed for blood sampling from the azygos vein in the future.

## 5. Conclusions

The levels of CTCs and TWIST (+) CTCs in blood from the azygos veins were significantly higher than those in the blood from the peripheral veins of patients with ESCC. However, with a limited number of patients included in this study, CTCs and TWIST (+) CTCs in the blood from the azygous vein were not associated with any clinicopathological factors. Therefore, further long-term, large-scale, and multicenter studies should be conducted to elucidate the role of CTCs in the blood from the azygos vein as predictive biomarkers for prognosis and chemotherapy responses in patients with ESCC.

## Figures and Tables

**Table 1:** Baseline clinicopathological characteristics of 40 patients with esophageal squamous cell carcinoma.

Characteristic	Value
Median age (years, range)	66 (51–79)
Sex, *n* (%)	
Male	35 (87.5)
Female	5 (12.5)
Location, *n* (%)	
Upper third	2 (5.0)
Middle third	27 (67.5)
Lower third	11 (27.5)
Median tumor size (cm, range)	3.4 (0.6–8.6)
Histology, *n* (%)	
Moderately differentiated	30 (75.0)
Poorly differentiated	10 (25.0)
Lymphatic invasion, *n* (%)	8 (20.0)
Vascular invasion, *n* (%)	3 (7.5)
T stage, *n* (%)	
T1	27 (67.5)
T2	6 (15.0)
T3	7 (17.5)
T4	0 (0)
N stage, *n* (%)	
N0	20 (50.0)
N1	13 (32.5)
N2	4 (10.0)
N3	3 (7.5)
M stage, *n* (%)	
M0	40 (100)
M1	0 (0)
Stage *, *n* (%)	
I	17 (42.5)
II	9 (22.5)
III	11(27.5)
IV	3 (7.5)

* based on the American Joint Committee on Cancer, 8th edition.

**Table 2:** Total and TWIST (+) circulating tumor cell counts in peripheral and azygos veins according to clinicopathological characteristics of 40 patients with esophageal squamous cell carcinoma.

Variable	Total CTC Count (/3 mL of Whole Blood)				TWIST (+) CTC Count (/3 mL of Whole Blood)			
	Peripheral Vein	*p*-Value	Azygos Vein	*p*-Value	Peripheral Vein	*p*-Value	Azygos Vein	*p*-Value
Age		0.716		0.648		0.761		0.472
<65 years	3.0 (0–20)		8.0 (0–42)		2.0 (0–20)		6.0 (0–40)	
≥65 years	3.0 (0–20)		7.0 (0–40)		2.0 (0–14)		6.0 (0–31)	
Sex		0.820		0.605		0.183		0.721
Male	3.0 (0–20)		7.0 (0–42)		3.0 (0–20)		6.0 (0–40)	
Female	3.0 (0–5)		6.0 (0–14)		1.0 (0–3)		6.0 (0–14)	
Tumor location		0.158		0.179		0.128		0.122
Upper third	8.0 (0–16)		17.5 (4–31)		7.0 (0–14)		17.5 (4–31)	
Middle third	4.0 (0–20)		8.0 (0–42)		3.0 (0–20)		6.0 (0–40)	
Lower third	2.0 (0–15)		4.0 (0–14)		1.0 (0–14)		2.0 (0–14)	
Tumor size		0.807		0.179		0.758		0.718
<3.4 cm	3.5 (0–15)		6.5 (0–32)		2.5 (0–14)		6.0 (0–27)	
≥3.4 cm	3.0 (0–20)		7.5 (0–42)		2.0 (0–20)		5.5 (0–40)	
Histology		0.788		0.778		0.963		0.914
Moderately differentiated	3.0 (0–20)		7.5 (0–42)		2.5 (0–20)		6.0 (0–40)	
Poorly differentiated	3.0 (0–16)		5.5 (0–32)		2.0 (0–14)		5.5 (0–31)	
Lymphatic invasion		0.238		0.170		0.325		0.174
Absent	3.0 (0–20)		7.5 (0–42)		2.0 (0–20)		6.5 (0–40)	
Present	1.5 (0–20)		4.5 (0–40)		1.5 (0–11)		3.5 (0–18)	
Vascular invasion		0.091		0.341		0.192		0.296
Absent	3.0 (0–20)		7.0 (0–42)		2.0 (0–20)		6.0 (0–40)	
Present	0 (0–3)		4.0 (0–14)		0 (0–3)		2.0 (0–8)	
T stage		0.069		0.121		0.219		0.124
T1	4.0 (0–20)		10.0 (0–42)		3.0 (0–20)		10.0 (0–40)	
T2	2.5 (0–15)		5.5 (2–19)		2.0 (0–14)		5.5 (0–15)	
T3	3.0 (0–3)		5.0 (0–14)		0 (0–3)		2.0 (0–8)	
N stage		0.719		0.886		0.965		0.499
N0	3.0 (0–14)		8.0 (0–42)		2.0 (0–13)		6.5 (0–39)	
N1	3.0 (0–20)		5.0 (0–36)		3.0 (0–20)		5.0 (0–36)	
N2	1.5 (0–16)		9.0 (0–31)		1.5 (0–14)		5.0 (0–31)	
N3	2.0 (0–20)		40.0 (4–40)		2.0 (0–11)		18.0 (2–40)	
Stage *		0.482		0.698		0.975		0.346
I	4.0 (0–14)		8.0 (0–42)		2.0 (0–13)		11.0 (0–39)	
II	3.0 (1–20)		6.0 (1–36)		3.0 (0–20)		6.0 (1–36)	
III	3.0 (0–16)		4.0 (0–31)		2.0 (0–14)		4.0 (0–31)	
IV	2.0 (2–20)		40.0 (4–40)		2.0 (0–11)		18.0 (2–40)	

Data are presented as median (range). * based on the American Joint Committee on Cancer 8th edition.

**Table 3:** Association between clinicopathological characteristics and circulating tumor cells from peripheral and azygous veins in 40 patients with esophageal squamous cell carcinoma.

Variable	Total CTC Count (/3 mL of Whole Blood)						TWIST (+) CTC Count (/3 mL of Whole Blood)					
	Peripheral Vein			Azygos Vein			Peripheral Vein			Azygos Vein		
	Low (<3) (*n* = 14)	High (≥3) (*n* = 26)	*p*-Value	Low (<7) (*n* = 19)	High (≥7) (*n* = 21)	*p*-Value	Low (<2) (*n* = 15)	High (≥2) (*n* = 25)	*p*-Value	Low (<6) (*n* = 19)	High (≥6) (*n* = 21)	*p*-Value
Age, *n* (%)			0.716			0.648			0.746			1.000
<65 years	5 (35.7)	10 (38.5)		12 (63.2)	13 (61.9)		5 (33.3)	10 (40.0)		7 (36.8)	8 (38.1)	
≥65 years	9 (64.3)	16 (61.5)		7 (36.8)	8 (38.1)		10 (66.7)	15 (60.0)		12 (63.2)	13 (61.9)	
Sex, *n* (%)			0.640			0.654			0.056			1.000
Male	13 (92.9)	22 (84.6)		16 (84.2)	19 (90.5)		11 (73.3)	24 (96.0)		17 (89.5)	18 (85.7)	
Female	1 (7.1)	4 (15.4)		3 (15.8)	2 (9.5)		4 (26.7)	1 (4.0)		2 (10.5)	3 (14.3)	
Tumor location, *n* (%)			0.205			0.494			0.057			0.494
Upper third	1 (7.1)	1 (3.8)		1 (5.3)	1 (4.8)		1 (6.7)	1 (4.0)		1 (5.3)	1 (4.8)	
Middle third	7 (50.0)	20 (76.9)		11 (57.9)	16 (76.2)		7 (46.7)	20 (80.0)		11 (57.9)	16 (76.2)	
Lower third	6 (42.9)	5 (19.2)		7 (36.8)	4 (19.0)		7 (46.7)	4 (16.0)		7 (36.8)	4 (19.0)	
Tumor size, *n* (%)			0.807			0.179			0.514			1.000
<3.4 cm	6 (42.9)	14 (53.8)		10 (52.6)	10 (47.6)		6 (40.0)	14 (56.0)		9 (47.4)	11 (52.4)	
≥3.4 cm	8 (57.1)	12 (46.2)		9 (47.4)	11 (52.4)		9 (60.0)	11 (44.0)		10 (52.6)	10 (47.6)	
Histology, *n* (%)			1.000			0.473			1.000			1.000
Moderately differentiated	11 (78.6)	19 (73.1)		13 (68.4)	17 (81.0)		11 (73.3)	19 (76.0)		14 (73.7)	16 (76.2)	
Poorly differentiated	3 (21.4)	7 (17.5)		6 (31.6)	4 (19.0)		4 (26.7)	6 (24.0)		5 (26.3)	5 (23.8)	
Lymphatic invasion, *n* (%)			0.416			0.120			0.444			0.120
Absent	10 (71.4)	22 (84.6)		13 (68.4)	19 (90.5)		11 (73.3)	21 (84.0)		13 (68.4)	19 (90.5)	
Present	4 (28.6)	4 (15.4)		6 (31.6)	2 (9.5)		4 (26.7)	4 (16.0)		6 (31.6)	2 (9.5)	
Vascular invasion, *n* (%)			0.276			0.596			0.545			0.596
Absent	12 (85.7)	25 (96.2)		17 (89.5)	20 (100)		13 (86.7)	24 (96.0)		17 (89.5)	20 (95.2)	
Present	2 (14.3)	1 (3.8)		2 (10.5)	1 (4.8)		2 (13.3)	1 (4.0)		2 (10.5)	1 (4.8)	
T stage, *n* (%)			0.548			0.893			0.550			0.406
T1	8 (57.1)	19 (73.1)		12 (63.2)	15 (71.4)		9 (60.0)	18 (72.0)		11 (57.9)	16 (76.2)	
T2	3 (21.4)	3 (11.5)		3 (15.8)	3 (14.3)		2 (13.3)	4 (16.0)		3 (15.8)	3 (14.3)	
T3	3 (21.4)	4 (15.4)		4 (21.1)	3 (14.3)		4 (26.7)	3 (12.0)		5 (26.3)	2 (9.5)	
N stage, *n* (%)			0.579			0.649			0.952			0.649
N0	6 (42.9)	14 (53.8)		8 (42.1)	12 (57.1)		7 (46.7)	13 (52.0)		8 (42.1)	12 (57.1)	
N1	4 (28.6)	9 (34.6)		8 (42.1)	5 (23.8)		5 (33.3)	8 (32.0)		8 (42.1)	5 (23.8)	
N2	2 (14.3)	2 (7.7)		2 (10.5)	2 (9.5)		2 (13.3)	2 (8.0)		2 (10.5)	2 (9.5)	
N3	2 (14.3)	1 (3.8)		1 (5.3)	2 (9.5)		1 (6.7)	2 (8.0)		1 (5.3)	2 (9.5)	
Stage *, *n* (%)			0.459			0.871			0.921			0.659
I	5 (35.7)	12 (46.2)		7 (36.4)	10 (47.6)		6 (40.0)	11 (44.0)		7 (36.8)	10 (47.6)	
II	2 (14.3)	7 (26.9)		5 (9.1)	4 (19.0)		3 (20.0)	6 (24.0)		4 (21.1)	5 (23.8)	
III	5 (35.7)	6 (23.1)		6 (45.5)	5 (23.8)		5 (33.3)	6 (24.0)		7 (36.8)	4 (19.0)	
IV	2 (14.3)	1 (3.8)		1 (9.1)	2 (9.5)		1 (6.7)	2 (8.0)		1 (5.3)	2 (9.5)	

* based on the American Joint Committee on Cancer 8th edition.

**Table 4:** Subgroup analysis for patients without a history of neoadjuvant chemotherapy.

Variable	Total CTC Count (/3 mL of Whole Blood)						TWIST (+) CTC Count (/3 mL of Whole Blood)					
	Peripheral Vein			Azygos Vein			Peripheral Vein			Azygos Vein		
	Low (<3) (*n* = 0)	High (≥3) (*n* = 18)	*p*-Value	Low (<7) (*n* = 14)	High (≥7) (*n* = 14)	*p*-Value	Low (<2) (*n* = 11)	High (≥2) (*n* = 17)	*p*-Value	Low (<6) (*n* = 14)	High (≥6) (*n* = 14)	*p*-Value
Age, *n* (%)			0.705			1.000			1.000			1.000
<65 years	4 (40.0)	9 (50.0)		6 (42.9)	7 (50.0)		5 (45.5)	8 (47.1)		6 (42.9)	7 (50.0)	
≥65 years	6 (60.0)	9 (50.0)		8 (57.1)	7 (50.0)		6 (54.5)	9 (52.9)		8 (57.1)	7 (50.0)	
Sex, *n* (%)			0.533			1.000			0.050			1.000
Male	10 (100)	15 (83.3)		12 (85.7)	13 (92.9)		8 (72.7)	17 (100)		13 (92.9)	12 (85.7)	
Female	0 (0)	3 (16.7)		2 (14.3)	1 (7.1)		3 (27.3)	0 (0)		1 (7.1)	2 (14.3)	
Tumor location, *n* (%)			0.185			0.648			0.022			0.648
Upper third	1 (10.0)	0 (0)		1 (7.1)	0 (4.8)		1 (9.1)	0 (0)		1 (7.1)	0 (0)	
Middle third	6 (60.0)	16 (88.9)		10 (71.4)	12 (85.7)		6 (54.5)	16 (94.1)		10 (71.4)	12 (85.7)	
Lower third	3 (30.0)	2 (11.1)		3 (21.4)	2 (14.3)		4 (36.4)	4 (5.9)		3 (21.4)	2 (14.3)	
Tumor size, *n* (%)			0.433			1.000			0.700			1.000
<3.4 cm	4 (40.0)	11 (61.1)		8 (57.1)	7 (50.0)		5 (45.5)	10 (58.8)		7 (50.0)	8 (57.1)	
≥3.4 cm	6 (60.0)	7 (38.9)		6 (42.9)	7 (50.0)		6 (54.5)	7 (41.2)		7 (50.0)	6 (42.9)	
Histology (n, %)			1.000			0.385			1.000			1.000
Moderately differentiated	8 (80.0)	13 (72.2)		9 (64.3)	12 (85.7)		8 (72.7)	13 (76.5)		10 (71.4)	11 (78.6)	
Poorly differentiated	2 (20.0)	5 (27.8)		5 (35.7)	2 (14.3)		3 (27.3)	4 (23.5)		4 (28.6)	3 (21.4)	
Lymphatic invasion, *n* (%)			1.000			0.596			1.000			0.596
Absent	9 (90.0)	15 (83.3)		11 (78.6)	13 (92.9)		10 (90.9)	14 (82.4)		11 (78.6)	13 (92.9)	
Present	1 (10.0)	3 (16.7)		3 (21.4)	1 (7.1)		1 (9.1)	3 (17.6)		3 (21.4)	1 (7.1)	
Vascular invasion, *n* (%)			0.357			1.000			0.393			1.000
Absent	9 (90.0)	18 (100)		13 (92.9)	14 (100)		10 (90.9)	17 (100)		13 (92.9)	14 (100)	
Present	1 (10.0)	0 (0)		1 (7.1)	0 (0)		1 (9.1)	0 (0)		1 (7.1)	0 (0)	
T stage, *n* (%)			0.464			1.000			0.612			0.396
T1	7 (70.0)	16 (88.9)		11 (78.6)	12 (52.2)		8 (72.7)	15 (88.2)		10 (71.4)	13 (92.9)	
T2	2 (20.0)	1 (5.6)		2 (14.3)	1 (7.1)		2 (18.2)	1 (5.9)		2 (14.3)	1 (7.1)	
T3	1 (10.0)	1 (5.6)		1 (7.1)	1 (7.1)		1 (9.1)	1 (5.9)		2 (14.3)	0 (0)	
N stage, *n* (%)			0.207			0.318			0.639			0.318
N0	6 (60.0)	11 (61.1)		7 (50.0)	10 (71.4)		6 (54.5)	11 (64.7)		7 (50.0)	10 (71.4)	
N1	2 (20.0)	7 (38.9)		6 (42.9)	3 (21.4)		4 (36.4)	5 (29.4)		6 (42.9)	3 (21.4)	
N2	1 (10.0)	0 (0)		1 (7.1)	0 (0)		1 (9.1)	0 (0)		1 (7.1)	0 (0)	
N3	1 (10.0)	0 (0)		0 (0)	1 (7.1)		0 (0)	1 (5.9)		0 (0)	1 (7.1)	
Stage *, *n* (%)			0.550			0.656			0.448			0.183
I	5 (50.0)	11 (61.1)		7 (50.0)	9 (64.3)		6 (54.5)	10 (58.8)		7 (50.0)	9 (64.3)	
II	2 (20.0)	5 (27.8)		4 (28.6)	3 (21.4)		2 (18.2)	5 (29.4)		3 (21.4)	4 (28.6)	
III	2 (20.0)	2 (11.1)		3 (21.4)	1 (7.1)		3 (27.3)	1 (5.9)		4 (28.6)	0 (0)	
IV	1 (10.0)	0 (0)		0 (0)	1 (7.1)		0 (0)	1 (5.9)		0 (0)	1 (7.1)	

* based on the American Joint Committee on Cancer 8th edition.

## Data Availability

The data that support the findings of this study are available from the corresponding author upon request.

## Supplementary Materials

The following supporting information can be downloaded at: https://www.mdpi.com/article/10.3390/cancers16162921/s1, Table S1: Comparison of circulating tumor cell counts between mid-esophagus and upper and lower esophagus; Table S2: Association between clinicopathological characteristics and circulating tumor cells from peripheral and azygous veins of 27 patients with mid-esophageal squamous cell carcinoma.
