# Supplementary material for: Clinical Implications of Circulating Tumor Cells in Patients with Esophageal Squamous Cell Carcinoma: Cancer-Draining Blood Versus Peripheral Blood
Source: Cancers (Basel). 2024 Aug 22;16(16):2921. doi: 10.3390/cancers16162921 (PMC11352898; doi:10.3390/cancers16162921)
Supplement: Supplementary file 1 [file cancers-16-02921-s001.zip › cancers-3149300-supplementary.pdf]

Supplementary Table S1. Comparison of circulating tumor cell counts between mid-esophagus and upper and lower esophagus.

Parameters	Peripheral vein			Azygos vein		
	Upper and lower esophagus (*n*=13)	Mid-esophagus (*n*= 27)	*p*-value	Upper and lower esophagus (*n*=13)	Mid-esophagus (*n*=27)	*p*-value
CTC count	2.0 (0–16)	4.0 (0–20)	0.308	4.0 (0–31)	8.0 (0–40)	0.124
TWIST (+) CTC count	1.0 (0–20)	3.0 (0–14)	0.064	2.0 (0–31)	6.0 (0–40)	0.106

Data are presented as median (range).

Supplementary Table S2. Association between clinicopathological characteristics and circulating tumor cells from peripheral and azygous veins of 27 patients with mid-esophageal squamous cell carcinoma.

Variable	Total CTC count (/3 mL of whole blood)						TWIST (+) CTC count (/3 mL of whole blood)					
	Peripheral vein			Azygos vein			Peripheral vein			Azygos vein		
	Low (<3) (*n* = 7)	High (≥3) (*n* = 20)	*p* value	Low (<7) (*n* = 11)	High (≥7) (*n* = 16)	*p* value	Low (<2) (*n* = 7)	High (≥2) (*n* = 20)	*p* value	Low (<6) (*n* = 11)	High (≥6) (*n* = 16)	*p* value
Age, *n* (%)			0.925			0.638			1.000			0.710
<65 years	3 (42.9)	8 (40.0)		5 (45.5)	6 (37.5)		3 (42.9)	8 (40.0)		5 (45.5)	6 (37.5)	
≥65 years	4 (57.1)	12 (60.0)		6 (54.5)	10 (62.5)		4 (57.1)	12 (60.0)		6 (54.5)	10 (62.5)	
Sex, *n* (%)			1.000			0.027			0.012			0.549
Male	6 (85.7)	18 (90.0)		8 (72.7)	16 (100)		4 (57.1)	20 (100)		9 (81.8)	15 (93.8)	
Female	1 (14.3)	2 (10.0)		3 (27.3)	0 (0)		3 (42.9)	0 (0)		2 (18.2)	1 (6.3)	
Tumor size, *n* (%)			0.649			0.069			1.000			0.696
<3.4 cm	4 (57.1)	11 (55.0)		8 (72.7)	7 (43.8)		4 (57.1)	11 (55.0)		7 (63.6)	8 (50.0)	
≥3.4 cm	3 (42.9)	9 (45.0)		3 (27.3)	9 (56.2)		3 (42.9)	9 (45.0)		4 (36.4)	8 (50.0)	
Histology (*n*, %)			1.000			0.206			0.633			0.675
Moderately differentiated	5 (71.4)	14 (70.0)		6 (54.5)	13 (81.3)		4 (57.1)	15 (75.0)		7 (63.6)	12 (75.0)	
Poorly differentiated	2 (28.6)	6 (30.0)		5 (45.5)	3 (18.8)		3 (42.9)	5 (25.0)		4 (36.4)	4 (25.0)	
Lymphatic invasion, *n* (%)			1.000			0.618			1.000			0.370
Absent	6 (85.7)	16 (80.0)		8 (72.7)	14 (87.5)		6 (85.7)	16 (80.0)		8 (72.7)	14 (87.5)	
Present	1	4		3	2		1	4		3	2	

	(14.3)	(20.0)		(27.3)	(12.5)		(14.3)	(20.0)		(27.3)	(12.5)	
Vascular invasion, *n* (%)			0.259			0.407			0.259			0.407
Absent	6 (85.7)	20 (100)		10 (90.9)	16 (100)		6 (85.7)	20 (100)		10 (90.6)	16 (100)	
Present	1 (14.3)	0 (0)		1 (9.1)	0 (0)		1 (14.3)	0 (0)		1 (9.1)	0 (0)	
T stage, *n* (%)			1.000			1.000			1.000			0.797
T1	5 (71.4)	16 (80.0)		9 (81.8)	12 (75.0)		5 (71.4)	16 (80.0)		8 (72.7)	13 (81.3)	
T2	1 (14.3)	2 (10.0)		1 (9.1)	2 (12.5)		1 (14.3)	2 (10.0)		1 (9.1)	2 (12.5)	
T3	1 (14.3)	2 (10.0)		1 (9.1)	2 (12.5)		1 (14.3)	2 (10.0)		2 (18.2)	1 (6.3)	
T4	0 (0)	0 (0)		0 (0)	0 (0)		0 (0)	0 (0)		0 (0)	0 (0)	
N stage, *n* (%)			0.289			0.138			0.123			0.138
N0	3 (42.9)	11 (55.0)		4 (36.4)	10 (62.5)		2 (28.6)	12 (60.0)		4 (36.4)	10 (62.5)	
N1	2 (28.6)	8 (40.0)		6 (54.5)	4 (25.0)		4 (57.1)	6 (30.0)		6 (54.5)	4 (25.0)	
N2	1 (14.3)	0 (0)		1 (9.1)	0 (0)		1 (14.3)	0 (0)		1 (9.1)	0 (0)	
N3	1 (14.3)	1 (5.0)		0 (0)	2 (12.5)		0 (0)	2 (10.0)		0 (0)	2 (12.5)	
Stage*, *n* (%)			0.625			0.541			0.283			0.248
I	2 (28.6)	10 (50.0)		4 (36.4)	8 (50.0)		2 (28.6)	10 (50.0)		4 (36.4)	8 (50.0)	
II	2 (28.6)	6 (30.0)		4 (36.4)	4 (25.0)		2 (28.6)	6 (30.0)		3 (27.3)	5 (31.3)	
III	2 (28.6)	3 (15.0)		3 (27.3)	2 (12.5)		3 (42.9)	2 (10.0)		4 (36.4)	1 (6.3)	
IV	1 (14.3)	1 (5.0)		0 (0)	2 (12.5)		0 (0)	2 (10.0)		0 (0)	2 (12.5)	

Note: *Based on the American Joint Committee on Cancer 8th edition.
